# Beliefs in Conflict: The Management of Teno Atlantic Salmon in the Sámi Homeland in Finland

**DOI:** 10.1007/s00267-020-01374-6

**Published:** 2020-11-05

**Authors:** Juha Hiedanpää, Joni Saijets, Pekka Jounela, Mikko Jokinen, Simo Sarkki

**Affiliations:** 1 Natural Resources Institute Finland (Luke), Turku, Finland; 2 University of Oulu, Oulu, Finland

## Abstract

The subarctic Teno River is one of the most significant spawning rivers for Atlantic salmon in Europe. In 2009, research indicated that the Teno salmon stock was in a weak state, and concern about the future of Atlantic salmon in the Teno River arose on both sides of the river, in Finland and Norway. In 2017, the governments ratified the new Teno fishing agreement (Teno Fishing Act 2017). The agreement aimed to reduce the fishing volume by 30%, and the new regulations concerned all users, including the indigenous Sámi, other locals, tourists, and fishing entrepreneurs. This triggered concern and anger in the Sámi community and among other locals generally. The dispute raised a question concerning the management of Teno salmon. We conducted a Q inquiry with 43 statements, covering aspects of interest, knowledge, management, and policy needs related to Teno salmon. We hypothesised that the key reason for the management tensions lay in how scientific and traditional knowledge fitted administrative knowledge requirements. By using self-organising maps (SOMs), four webs of beliefs emerged from the data: traditional Sámi fishing; salmon protection; equal economic opportunity; and evidence-based decision-making. We also further analysed the statements according to how they reproduced diverging and similar beliefs. We discuss the identity-related struggle, rights, and stakes and the underlying issue of confidence and respect.

## Introduction

Environmental resources underpin human wellbeing. However, ensuring equal wellbeing is challenged by the diverse positions and perspectives of local actors, scientists, and administrators in the face of resources, their use, and management (e.g., Diaz et al. 2018). The acceptability of policy and management decisions can be only partly measured by objective yardsticks. They are also defined by actors’ perceptions, which are grounded in the experience and habits of thinking that condition beliefs regarding the future (Hiedanpää and Bromley 2016, p. 55–72). In our paper, we address the beliefs in resource contestation to better understand which aspects of knowledge production, management, and policy are considered successful and failures, and pinpoint crucial social, cultural, and political features institutional design processes need to take into an account. We focus on the Teno salmon and its management as a case study.

**Fig. 1:**
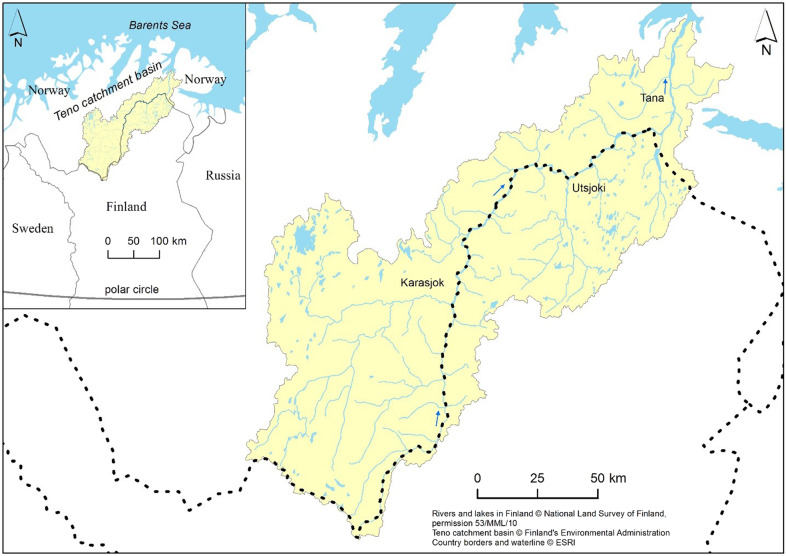
The Teno River catchment basin is located between Finland and Norway

Migratory fish have provided nourishment for local cultures in the Circumpolar North, created permanent human settlements, and provided livelihoods (Autti 2017). The subarctic Teno River is one of the most significant spawning rivers for Atlantic salmon in Europe. It is one of the few remaining large watersheds that still support abundant Atlantic salmon stock, with little or no human impact on the system except for fishing (Erkinaro et al. 2019). In addition to the Teno River itself, the watershed consists of ~30 tributaries, which support genetically distinct salmon populations (Vähä et al. 2017). The total length of the watershed is around 330 km, of which 270 km run along the border of Finland and Norway. The Teno is called the Tana in Norwegian and the Deatnu in Northern Sámi (Fig. 1).

It is also in the heart of the Sámi communities that share a border between Finland and Norway, flowing through three central Sámi municipalities Deatnu (No: Tana) and Kárášjohka (No: Karasjok) in Norway, and Ohcejohka (Fi: Utsjoki) in Finland. The Sámi people of the Teno River call themselves *čáhcegátte olbmot* in Northern Sámi, meaning people living by the shore, and they have fished in the region since time immemorial (Solbakk and Muladal 2007, 12, and 16–17). It is because of the wild Atlantic salmon that there are human settlements in the Teno valley (Helander 1985; Helander-Renvall and Markkula 2011). Salmon have provided people with important sustenance and income, along with farming (Pieski 2013) and other nature-based occupations practised in accordance with the natural cycles (Lehtola 2012, p. 38).

In 2009, research findings indicated that the Teno salmon stock was in a weak state, and concern about the future of Atlantic salmon in the Teno River arose because of a decline in the number of spawners on both sides of the river in Norway and Finland (Niemelä et al. 2009). A growing body of research provides policy advice on how to manage wild Atlantic salmon. Previously, the research focused on biological aspects of the salmon population and its lifecycle. The development of genetic analyses (genetic stock identification (GSI)) in recent years has made it possible to identify 30 different salmon stocks in the Teno watershed (Niva et al. 2016, p. 9). A stock-specific knowledge of the stocks in the upper part of the rivershed, which was faring poorly, played a significant role in the negotiation of the new Teno fishing agreement (Niva et al. 2016, p. 20; Vähä et al. 2017).

The right to fish for salmon in the Teno in Finland is tied to real estate ownership by the river, not ethnicity (Fishing Act 379/2015; Real Estate Formation Act 554/1995). Three major groups hold fishing rights: (1) local real estate owners along the Teno river, mainly the local Sámi people; (2) real estate owners who do not live in the watershed area, either Sámis and Finns or cabin owners, and are eligible to buy a quoted (max one-third of 11,000) cheap shore or boat fishing licence; (3) non-local real estate owners, both non-Sámi and Sámi, whether or not they have a cultural connection with salmon fishing in the Teno. In addition to these rights holders, local residents can buy inexpensive seasonal fishing permits. The quota for local residents differs markedly from that for non-local residents. Tourists can buy day-based fishing permits. The number of permits is restricted and sold by the state (Turunen et al. 2020). In Norway, the right to fish is tied to farming (Lovdata 2014). In 2016, the governments of Finland and Norway signed a new fishing agreement, which replaced the previous one from 1990.^1^ The agreement was ratified and entered into force on 1 May 2017, and it aimed to reduce the fishing volume by 30%. The new regulations concerned all users—the indigenous Sámi, other locals, tourists, and fishing entrepreneurs. This triggered concern and anger in the Sámi community, among other locals and non-local real estate and cottage owners. In the Northeast Atlantic area, Europe’s only indigenous people, the Sámi, have long relied on salmon for a significant portion of their sustenance. Salmon fishing is the foundation of the Sámi river culture and identity (Joks and Law 2017; Law and Joks 2019). In addition, the livelihoods of some non-Sámi people, whether they are water use right holders or tourism entrepreneurs, greatly depend on the Teno salmon (Holmberg 2018). Sámi activists have demonstrated, saying that indigenous Sámi rights to salmon should come first (Yle 2017). The Sámi Parliament lodged a complaint against the Ministry of Agriculture and Forestry in Finland for not fulfilling its obligation to negotiate with the Sámi Parliament during the preparation of the agreement, as the Act on the Sámi Parliament (974/1995) requires. The Sámi Parliament requested that the Finnish Chancellor of Justice take note that the law had been violated (Sámi Parliament 2016). In addition, the Teno has been the most popular and iconic recreational and tourist salmon fishing river in Finland since 1800 (Kojo 1983; Pokki et al. 2018; Solbakk and Muladal 2007). In Norway, the river is not a tourist destination.

There is a long history of contradictions between stakeholders and complex management issues in determining the access of various fisher groups to the Teno salmon fisheries; our case is just one episode. In every Teno fishing agreement since 1873, the fisher group has had to give up their access to fish, either through the narrowing of fishing times or changes in fishing equipment and fishing areas. These regulations have always affected Sámi culture, nature-based sustenance, and communities on both the Finnish and Norwegian sides of the river (Pedersen 1986; Pieski 2000, p. 49). The first agreement already constituted the end of Sámi self-governance (Solbakk 2003, 2011), ending many important treaties and agreements regarding fishing between local Sámi from both sides of the river (Burgess 1996).

The Teno conflict is one example of conflicts over natural resources in the Sámi homeland emerging from the unresolved land and water rights of the Sámi people. The conflicts involve mining controversies (Lassila 2018), forestry and reindeer herding (Jokinen 2014), nature conservation, and reindeer herding (Heikkinen et al. 2010). Fisheries conflicts have also been widely examined in Canada (Denny and Fanning 2016; Young et al. 2018), Scotland (Butler et al. 2015), and Norway and Finland (Brattland and Mustonen 2018). These conflicts can often be traced back to unresolved land and water rights, perceived colonialism, different ways of life, problems in policy participation, and the human rights violations experienced by the Sámi people.

The current dispute has raised the question of fairness in Teno salmon management, and the cultural, social, and economic aspects of the sustainable management of the Teno salmon populations have become a topical issue. What was first a biological salmon management problem has become a complex policy problem, encompassing cultural, social, economic, and administrative issues. This was also the impetus for us to study the underlying habits of thinking constituting the Teno conflict, and the societal structures and functions they reveal.

We conducted a Q inquiry, with 43 statements covering aspects of interest, knowledge, management, and policy needs related to the Teno salmon. The practical added value of this paper is to generate knowledge that can inform policy planning by identifying the nuanced aspects of interests underlying salmon contestation. This paper has added methodological value, because it presents a case study in which the Q method is used to identify the role and significance of shared habits of thinking in the formulation and implementation of salmon policy. The paper has the theoretical added value of revealing a still invisible functioning of the conflict and offering pathways for real-life conflict resolution.

## Perspective, Materials, and Method

### Habits and Beliefs

Methodologically, our key concepts focus on habits and beliefs. For pragmatist philosophers such as John Dewey (1988) and Charles S. Peirce (1934), a habit is a general disposition, not a repetition. Habits are a spectrum of potential feelings, actions, and thoughts. All thoughts are grounded in habits of thinking that are the product of earlier volitional acts or social and environmental conditioning (Sheriff 1993). As Peirce (1934, vol. V, note 398) explicates: “The essence of belief is the establishment of a habit; and different beliefs are distinguished by the different modes of action to which they give rise.” Our task is to identify these beliefs and explicate how they give form to salmon management problems, the explicit mechanisms and nuances of which remain invisible and poorly understood. Our analysis is abductive (Niiniluoto 2018)—that is, from a real-life phenomenon, we identify habits of thinking and infer what these habits are about. We are not interested in single items of individual opinions, but in the clusters of beliefs. We therefore applied the factor analysis known as the Q method (Watts and Stenner 2012).

### Qualitative Interviews

We began with qualitative interviews. We interviewed 39 local fishers in the summers of 2015 and 2016.^2^ The semi-structured thematic interviews (Huntington 1998) concerned local traditional knowledge, and its transmission and use in salmon management on the Finnish side of the Teno valley. The study area covered almost the entire river, from the upper to the lowest parts up to the Norwegian border. Based on the interviews, a wide range of experiences and opinions about salmon management was collected. This information was later used to create the concourse for the Q set of this study.

### Q Method

We selected the Q method to identify the habits of thinking underlying tensions in Teno salmon management and policy. The Q method is used in the social sciences to study respondents’ views of statements. The name “Q” comes from the form of factor analysis used to find correlations between statements across a sample of subjects. A Q sort is a ranking of statements printed on small cards that form a Q sort grid table (Q board, Appendix 3). The general use of statements’ ranking is intended to capture the idea that respondents think about the given statements in relation to other statements, rather than in isolation.

The statements covered interests, knowledge, management, and policy. Initially, we designed some 90 statements, of which 43 were chosen for the final Q set. All our four empirical concepts received quite equal coverage. The statements were designed in Finnish and then translated into Northern Sámi. Translation was crucial, because most Sámi fishers’ native language is Northern Sámi. The participants could choose the language in which they wished to participate (Finnish or Northern Sámi).

In 2018, we held two workshops (one at the Reindeer and Fisheries Days of Lapland and the other at the annual Teno Info event), where we collected quantitative and qualitative data. Although the total number of respondents was quite low (*N* = 23), we achieved good coverage of the relevant people within the study. Clustering methods that have been shown to be robust with respect to sample size (Kiang et al. 2005) were also used here to cluster respondents. The participants were self-selected. We did not know which local Sámi and non-Sámi people would attend the workshops. Their participation was not randomised, but nor was it hand-picked. Some of the attendees had an interest which had motivated them to participated in the Teno Info meeting, which was an open meeting for all local salmon cooperative members. Altogether, in two workshops, relevant salmon scientists actively participating in Teno salmon management were present (4), as well as regional and national administrators (3). The participants could indicate which group they represented: a Sámi fishing group, administration, science, or a local non-Sámi or tourism group. Of all the respondents, three participants were women. Most participants were Sámi and non-Sámi locals. Participating scientists and administrators covered all those primarily working on Teno salmon issues.

The first workshop was held during the Reindeer and Fisheries Days of Lapland conference in Lapland in May 2018. The event was for national and regional-level actors, administrators, and representatives of interest groups. We held a session with five self-selected people involved in salmon governance, and therefore interested in the wild Atlantic salmon and its management success. One participant with no interest in salmon, salmon fishing, or its management was excluded from the dataset.

The second workshop was arranged during the Teno Info event in Utsjoki in mid-May 2018. Salmon scientists and the fisheries administration meet the Teno River fishers, landowners, and interested stakeholders annually. The Teno Info event concerns the current state of the salmon and latest managerial updates. Thirty participants attended the meeting, of which 17 participated in our dataset. Most of the older Sámi participants refrained from participating in our dataset.

In addition to the two workshops, our sample was complemented by three more participants from the upper parts of the watershed in the villages of Karigasniemi and Outakoski, who were invited to participate in the Q sorting phase.

During the Q sorting phase, we asked the informants to sort the cards according to how much they agreed or disagreed with the statements written on each card. The scale was from −3 (most disagree) to +3 (most agree). If a person strongly agreed with a statement, the card was placed in the +3 section; if the respondent strongly disagreed, it was placed in the −3 section. Fewer places were available for the strongest agreements and more for the middle course, corresponding to neutral opinions. This means the respondents were forced to sort the statements into a quasi-normal distribution. It also means that the sorters had to arrange the statements in relation to each other following the quasi-normal distribution (Eyvindson et al. 2015).

### Statistical Analyses

A self-organising map (SOM; Kohonen 1982; 2001) was used to cluster participants based on the Q sorting for each individual. In general, an SOM is an unsupervised (i.e., no “right answer”; no underlined label; no human supervision) dimensionality reduction method that visualises high-dimensional data (here: 43 claims) in a low-dimensional (typically two-dimensional) map. To facilitate a quantitative analysis of the map and the Q sorting data, similar participants were grouped—that is, clustered. A cluster separation (of participants) that both minimised the intra-cluster distance (similar responses) and maximised the inter-cluster distance (distinct responses) was selected. This two-stage procedure, which first uses an SOM to produce the prototypes and then clusters the prototypes in the second stage, has been found to perform well compared with direct clustering of the data and to reduce the computation time (Vesanto and Alhoniemi 2000). The two SOM dimensions were clustered using a k-means algorithm (Kohonen 2014), and the Davies Boulding validity index (Davies and Bouldin 1979) was used as a performance criterion. In the parameter optimisation, the SOM net sizes (x and y dimensions), initial learning rate, and number of clusters (i.e., parameter k of the k-means algorithm) were altered using a grid search until the minimum of the Davies Boulding index was found based on the elbow criterion. Each trial SOM consisted of 1000 training rounds, and the learning rate function was the inverse-of-time, which ensures that all the input samples have an approximately equal influence on the training result. The statistical analyses were performed using RapidMiner software (version Studio Large 9.0.003.; Mierswa et al. 2006).

## Results: Webs of Beliefs

### Clusters

**Fig. 2:**
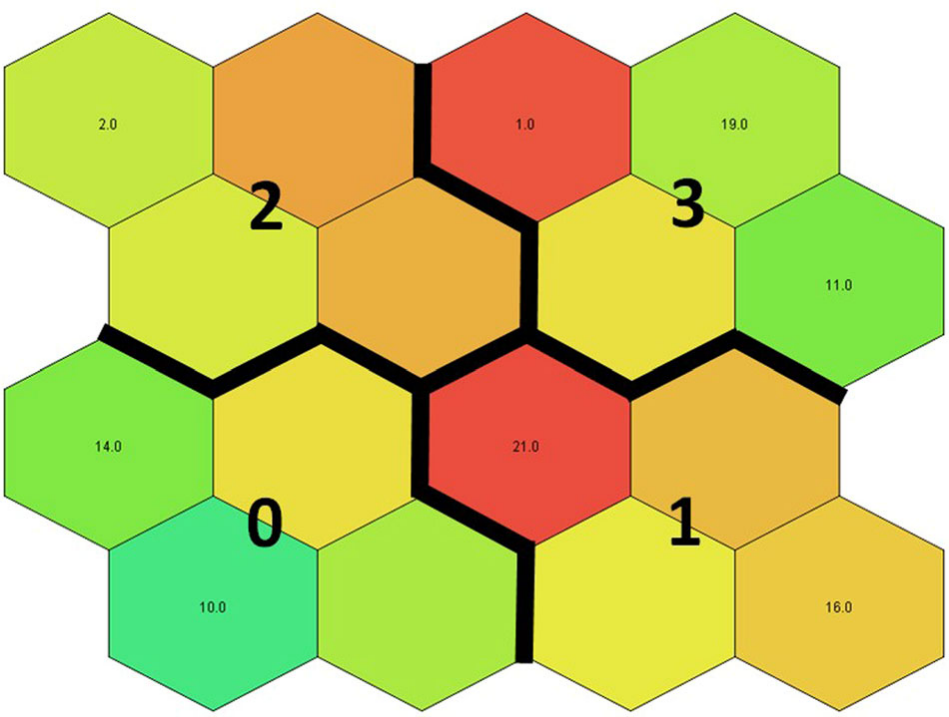
General distribution of clusters

**Fig. 3:**
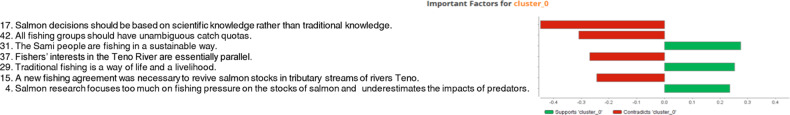
Important factors for Cluster 0: traditional Sámi fishing

**Fig. 4:**
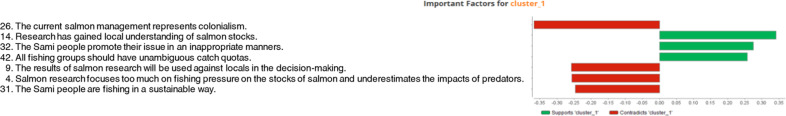
Important factors for Cluster 1: salmon protection

**Fig. 5:**
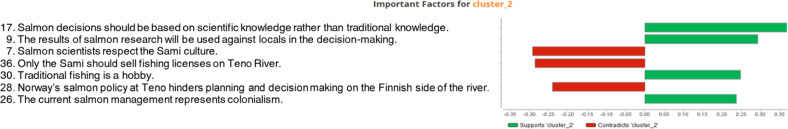
Important factors for Cluster 2: equal economic opportunity

**Fig. 6:**
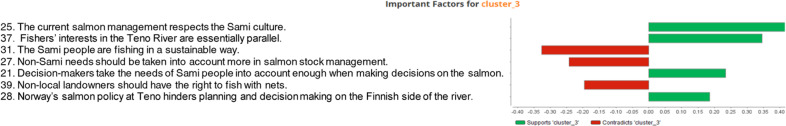
Important factors for Cluster 3: evidence-based decision making

The best-performing SOM model suggested four respondent clusters. In this section, we describe the clusters with the statements that most characterise each cluster (created by the SOM). The clusters we identified were: traditional Sámi fishing (Cluster 0); salmon protection (Cluster 1); equal economic opportunity (Cluster 2); and evidence-based decision making first (Cluster 3), Figs. 2–6. (see all 43 statements in the SOM heat-maps separated into aspects of interest, knowledge, management, and policy needs related to the Teno salmon, Appendix 2).

#### Cluster 0—traditional Sámi fishing

For Cluster 0, traditional local knowledge was in most agreement with sustainable salmon management. Accordingly, there was a conflict of interest between traditional Sámi fishing and other fishers, and between scientific knowledge concerning salmon and local knowledge concerning the Teno River. Cluster 0 postulated that the scientific approach focuses on fishing pressure and insufficiently on predators, the wider river environment, or salmon lifecycle. Salmon fishing was seen as a traditional livelihood, not a hobby. Sámi fishers had a particular relationship with the Teno River, which was inherently different from those of the non-Sámi. People from outside the Sámi culture therefore should not have a similar standing in salmon management to the Sámi concerning the Teno River. For Cluster 0, the divide of “us” as legitimate insiders and “them” as outsiders was essential.

#### Cluster 1—salmon protection

For Cluster 1, science provided the best grounds for sustainable decisions, not traditional Sámi knowledge. The managerial focus should be strictly on salmon. The task was to provide management rules, not to oppress people. Furthermore, the view was that the state had not colonised Sámi fishers. Policy was needed to avoid maladaptation, and the policy should be designed formally and based on strictly defined quotas. The view of this cluster was that sustainability must not be compromised.

#### Cluster 2—equal economic opportunity

Cluster 2 built on the beliefs in the importance of scientific advice in policymaking, but the cluster reflected the view that scientific advice was misused within the colonial system of governance. However, in this cluster, individual scientists were seen as respecting Sámi culture. Salmon management on the Norwegian side was not blamed, because it was seen as working better there, and salmon fishing should not be an economic privilege of the Sámi. Sámi fishing rights, trust in science and scientists regarding traditional knowledge and its continuity, salmon management through sustainable fishing, and the significance of salmon for local entrepreneurs also played a significant role in the comments.

#### Cluster 3—evidence-based decision making

Cluster 3 built on a shared belief that evidence-based policy respected the Sámi, but the beliefs in the cluster opposed all types of fishing pressure on salmon, whether it was caused by the Sámi, non-Sámi locals, or landowners living outside the river basin. This cluster’s view was that there should be no further issuance of fishing rights, but the current Sámi rights and future should be respected. The interests were essentially the same: in their own ways, beliefs in this cluster showed care for the fish. However, it was felt in this cluster that the Norwegians were too loose with issuing fishing permission, and that local fishing habits were unsustainable.

### Divergence and Similarity

In this section, we present statements that show divergence or similarity in SOM heat-maps (Appendix 1). Colder clusters (towards blue) are more in disagreement with the statement; warmer ones (towards red) are more in agreement (see Appendix 2 for all the statements).

#### Interests

**Fig. 7:**
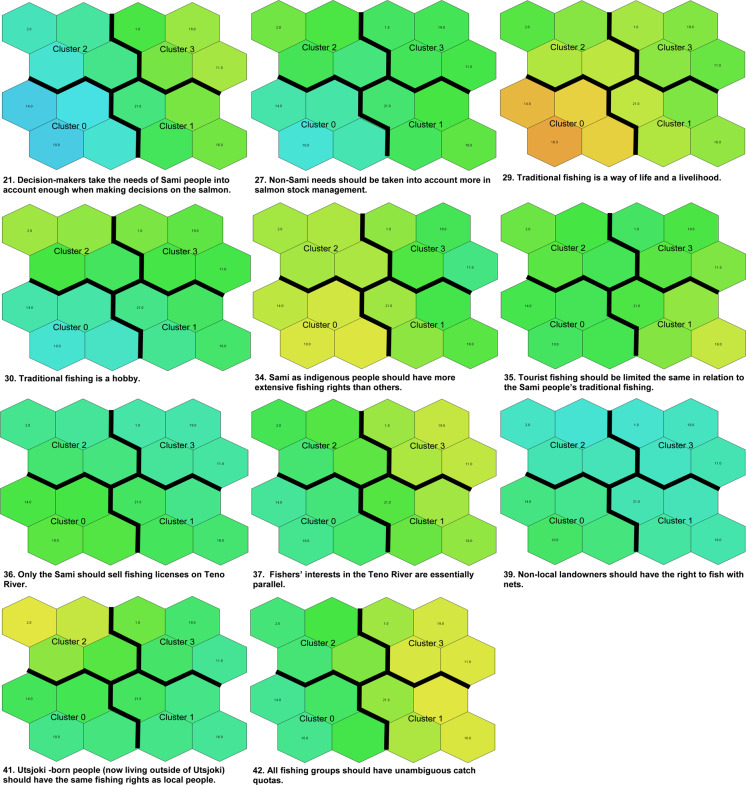
SOM heat-maps of interests Q statements 21, 27, 29, 30, 34, 35, 36, 37, 39, 41 and 42. A more blueish color represents disagreement and a more reddish color represents agreement in stakeholder clusters 0 – 3 separated by thick black lines

The evidence-based decision-making cluster indicated that *fishers’ interests in the Teno River were essentially parallel* (see Appendix 2, statement: 37; Appendix 1, Fig. 7). It was the fish and their sustainable catch that mattered. However, the traditional Sámi fishing cluster did not share this optimism: it quite strongly opposed the idea that different fishers in the Teno shared an underlying interest. The evidence cluster saw this in more general terms, while the traditional Sámi fishing cluster saw it as a concrete issue of the need to protect their rights to traditional net fishing methods (such as weir and drift netting, which were still allowed). Local needs differed from those held by scientists, administrators, and policy planners. In the traditional Sámi fishing cluster, fishing was a primary need, an essential constituent of individual and social wellbeing. Furthermore, it was clear for them that this was a matter of ethnic and cultural self-determination (on self-determination see Nuttall 2019). For scientists and policy planners, the need was more of a technical obligation related to decision-making procedures. The need concerned the fulfilment of the institutional requirements.

In the face of conflict, the traditional Sámi fishing cluster strongly agreed with the statement that decision-makers *did not sufficiently take Sámi needs into account* (statement: 21), while the evidence cluster held that they in fact did. This represented a conflict of interest concerning the substantive and procedural decision-making issues. Except for the evidence cluster, the other clusters slightly held that as indigenous people, *the Sámi people should have more extensive fishing rights than other fishers* (34). In other words, there should be positive discrimination for Sámi people in relation to salmon fishing. The Convention of Biological Diversity (CBD 2004, 2020) prescribes taking indigenous rights (knowledge, practices, and innovations) into account when species protection and indigenous rights are present in the same situation.

If the objective of the Teno fishing agreement was to reduce fishing pressure, it was impossible to sustain the *status quo* of all the fishing rights in Teno salmon management. If the cut of 30% was not taken from the Sámi catch, it must be reduced from the other rights. The general view, and especially in the traditional Sámi fishing cluster, held that *non-Sámi local interests* should not weigh more than they currently did in planning and decision making (27). All clusters agreed that *non-local landowners* should not be allowed to use nets for fishing (39). Only the equal opportunity first cluster quite strongly agreed with the statement that the *tourists’ allowable catch* should be restrained in the same proportion as the Sámi fishers’ allowable catch (35). The cut should be taken from others’ catches. Procedurally, the traditional Sámi fishing cluster held that there should not be *a strict quota* for all fisher groups (42). The Sámi should be protected from such a quota. Only the equal economic opportunity cluster supported the statement that *those born* by the Teno River should have the same rights as those who still lived there (41). The equal economic opportunity cluster did not oppose fishing, but they were unwilling to positively discriminate on behalf of the Sámi. All the clusters diverged from the statement concerning whether only the *Sámi should be entitled to sell fishing licences* (36).

For the traditional Sámi fishing cluster, traditional fishing was *not a hobby* (30), but a *livelihood and enterprise* (29). However, the equal economic opportunity cluster saw traditional fishing more as a hobby. This might have been because the habit of thinking did not encompass traditional net fishing, despite the fact that the entrepreneurs constituting the cluster might have this right: they might be focused on attracting more tourist fishers to the river in the summer and getting them to use their services (accommodation, food, guiding, etc.). The evidence cluster and the equal economic opportunity cluster were slightly in favour of seeing Sámi fishing as a livelihood and enterprise.

#### Knowledge

**Fig. 8:**
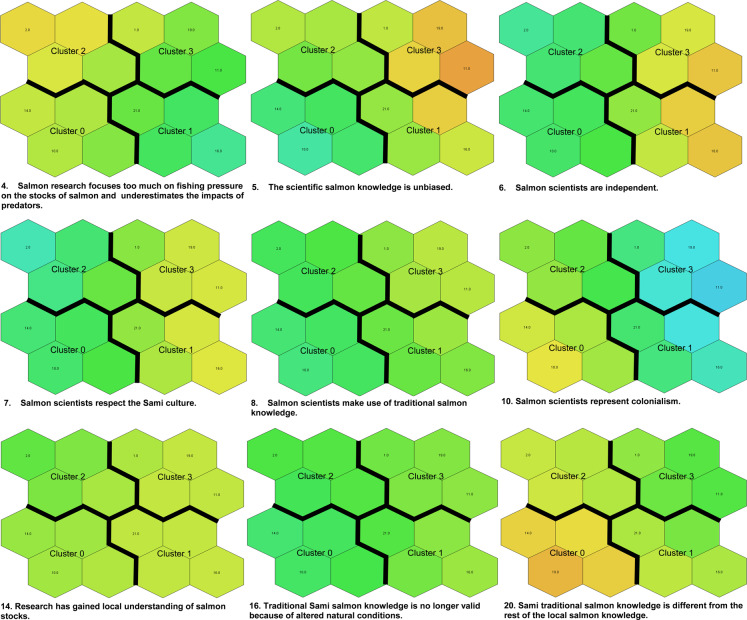
SOM heat-maps of knowledge Q statements 4, 5, 6, 7, 8, 10, 14, 16 and 20. A more blueish color represents disagreement and a more reddish color represents agreement in stakeholder clusters 0 – 3 separated by thick black lines

In general, the local fishers had serious issues with salmon science. However, it was especially evident that science and scientists were not viewed in the same way (Appendix 1, Fig. 8). According to the traditional Sámi fishing cluster, *salmon science was biased* (5); the equal economic opportunity cluster believed this even more strongly, indicating that *salmon scientists were not neutral* (6), but in fact served particular societal interests. The traditional Sámi fishing cluster extended this idea, holding that *salmon scientists represented colonialism* (10). The salmon protection and evidence clusters diverged from this view. For them, salmon *scientists respected* Sámi culture (7), and the evidence cluster held that *salmon scientists utilised traditional knowledge* (8) in their work. The equal economic opportunity cluster built on scepticism concerning the respect scientists showed for Sámi traditions.

All the clusters supported the statement that salmon science had *increased the local understanding* of salmon issues (14). This view was supported by the fact that local fishers had collected scale samples for 40 years, and local stakeholders had been invited to the annual Teno Info event, where population and lifecycle issues had been discussed for several years. In addition, many locals had continuous contact with salmon scientists outside this event—for example, if they caught a strange fish, they contacted fishery scientists for advice and further research. Meanwhile, some scientists enjoyed good relations with local fishers outside this collaboration. Social learning had evidently taken place in recent decades.

However, not all local knowledge was considered the same. The traditional Sámi fishing cluster strongly indicated that *traditional knowledge differed* from other local knowledge (20). The salmon protection and the equal economic opportunity clusters tended to build on this belief as well, but the evidence-based decision-making cluster did not.

The traditional Sámi fishing cluster challenged the statement that *traditional knowledge no longer applied* in changing environmental conditions (16), while the salmon protection and the evidence clusters were slightly in favour of this. For the traditional Sámi fishing and equal economic opportunity clusters, salmon science focused too much on *fishing pressure and belittled the effects of predators* on salmon (4).

#### Management

**Fig. 9:**
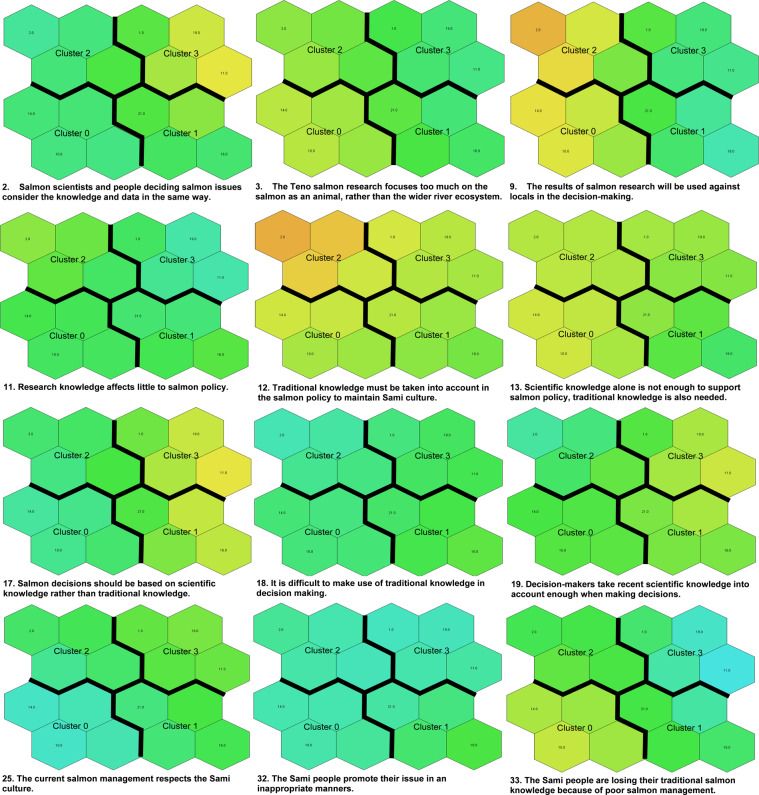
SOM heat-maps of management Q statements 2, 3, 9, 11, 12, 13, 17, 18, 19, 25, 32 and 33. A more blueish color represents disagreement and a more reddish color represents agreement in stakeholder clusters 0 – 3 separated by thick black lines

The traditional Sámi fishing cluster saw *bad salmon management* as the root cause of the loss of traditional Sámi knowledge (33) (Appendix 1, Fig. 9). The evidence cluster disagreed with the claim that the Sámi people were losing traditional knowledge because of management. Instead, this cluster built on the belief that the management *showed respect* for the Sámi fishing culture (25).

For the equal economic opportunity cluster, *local traditional knowledge should be incorporated better* in decision making to have a positive influence on Sámi culture (12). Why did the Sámi rights cluster not support this? Perhaps the question of Sámi engagement is in itself too colonial.

All but the salmon protection cluster were slightly in favour of the claim that *scientific knowledge was insufficient* for decision making, but traditional knowledge was also needed (13). This cluster believed in science more than the others. The statement that decisions should be based on *scientific knowledge rather than traditional knowledge* (17) separated the traditional Sámi fishing cluster from the others. The evidence cluster agreed that *scientific knowledge influenced decision making* (11). The others perceived this statement as lukewarm. The claim that the *Sámi fishers promoted their interests* inappropriately (32) only received slight support from the equal economic opportunity cluster.

The evidence cluster also agreed that *scientists and decision-makers understood* salmon-related knowledge and formal knowledge requirements similarly (2), while the others, especially the equal opportunity cluster, slightly opposed this. The evidence cluster also believed that *decision-makers incorporated* the most recent scientific knowledge sufficiently (19). The equal economic opportunity cluster opposed this slightly, believing it would not be especially *difficult to apply local traditional knowledge* in Teno salmon management planning and decision making (18), while the others saw it as a difficulty.

It was slightly shared by all clusters that salmon-related science concentrated too much on *salmon and not on the wider Teno River* environment (3). However, the evidence cluster would not go so far as to claim that *salmon science was used against local people* (9). This latter statement divided the clusters into two joint clusters, traditional Sámi fishing and equal economic opportunities on the one hand, and salmon protection and evidence clusters on the other.

#### Policy

**Fig. 10:**
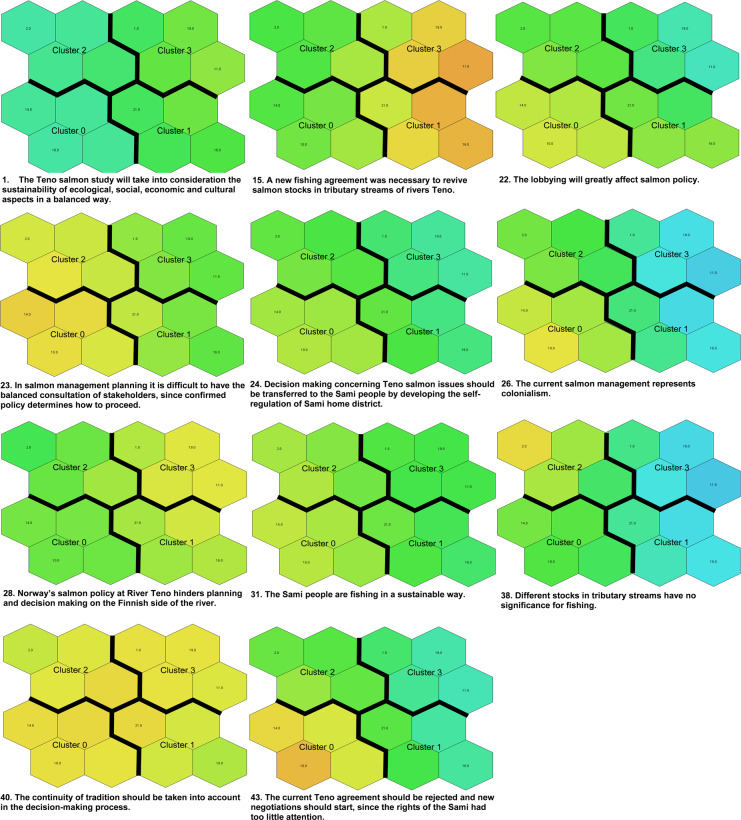
SOM heat-maps of policy Q statements 2, 3, 9, 11, 12, 13, 17, 18, 19, 25, 32 and 33. A more blueish color represents disagreement and a more reddish color represents agreement in stakeholder clusters 0 – 3 separated by thick black lines

The evidence-based decision-making cluster held that the fishing pressure on the Teno River negatively affected how *salmon subpopulations* in tributaries were doing (38), and a *new fishing agreement* was therefore necessary to revive the Teno salmon subpopulations (15) (Appendix 1, Fig. 10). However, there was a division concerning how fishing was understood to affect the different salmon subpopulations. Locals, especially the equal economic opportunity cluster, held that fishing did not greatly affect the situation. Therefore, in this view, the current *Teno fishing agreement was unnecessary* and should be cancelled, because it was obsolete, and the negotiation process should be restarted (43).

The traditional Sámi fishing and equal economic opportunity clusters held that *Sámi fishing was sustainable* (31). The evidence and the salmon protection clusters recognised the negative influence of fishing. In all, the traditional Sámi fishing cluster seemed to hold that tourists negatively impacted the salmon population, while the equal opportunity cluster did not indicate there was a problem with the state of the salmon population, and if there was a problem, it was because of predation in the sea and river.

Both the equal economic opportunity and evidence clusters agreed with the statements that *Norwegian salmon policy* practices negatively affected practices in Finland (28). Finland had little to say on how the Norwegians fished on the first 60 km of the Teno River, which ran on their side. The regulation had been quite similar on both sides of the river since the 2017 agreement. The traditional Sámi fishing cluster did not blame the Sámi fishers of Norway. For the salmon protection cluster, the solution should be the restriction of fishing, no matter from where the pressure emanated.

The evidence and salmon protection clusters opposed the claim that salmon *governance was colonial* (26). The conflict was not one of interests alone. It also concerned human and indigenous rights, and the substantial and procedural legal principles of natural resource planning and decision making. The state of Finland played an active role in how these principles were enacted, and how these rights were implemented (see Heinämäki et al. 2017).

The salmon protection and equal economic opportunity clusters were somewhat indifferent to *Sámi self-governance*. Neither the traditional Sámi fishing nor the evidence cluster agreed strongly with the question of whether Sámi self-governance should be developed further in relation to salmon management (24). The first mildly favoured it, while the latter disfavoured it slightly. Perhaps this question about the basic institutional structure was too abstract in this case-specific context.

In management planning and decision making, it was difficult to hear different stakeholders equally, because the *chosen policy* predetermined which actions were possible, and which were not (23). Once the salmon protection was decided, and especially when the decision to cut the fishing pressure by 30% had been made, there was no leeway for further action. The traditional Sámi fishing and equal economic opportunity clusters agreed strongly with this claim, while the salmon protection and evidence clusters had only moderate feelings about it. However, the traditional Sámi fishing cluster slightly believed that *lobbying pressure* influenced decision making (22), whereas the other clusters did not.

Indeed, there was little need for policy adaptation, because all were lukewarm about the statement that all *three sustainability realms* (ecological, economic, and social) were well incorporated in salmon planning and decision making (1). In addition, all clusters agreed with the statement that the *continuity* of the Sámi fishing culture should be taken into account in salmon policy and management planning and decision making (40).

## Discussion

The clustered beliefs point to wider questions at play in the Teno case, forming the basis of salmon policy and management problems. By following the abductive logic here, we discuss *a case* that has hitherto been invisible and unarticulated, but which has now been revealed by the Q study.

### Rights and Stakes

In sustainability policy and science, it is commonly considered that all stakeholders affecting or affected by decisions should be recognised and given an opportunity to participate in policy planning and decision making (Reed et al. 2009). However, this perspective is often blind to the existing property rights, power asymmetries, structural oppression, and discrimination of indigenous people (Ojha et al. 2010; FAO 2016; Banerjee 2000; von der Porten and de Loë 2014). According to the clustering of our statements, we propose a distinction between stakes and rights.

Concerns about rights and how they are exercised emerge from our results. The clustered beliefs represent different ideas about legal and social positions in management. The equal economic opportunity cluster seems to promote equal opportunities for all actors. This represents a relatively all-inclusive approach to the identification of stakeholders. The evidence cluster promotes the consideration of evidence first—that is, it is connected with evidence and science-based decision making in which scientific knowledge can justify decisions, even if some stakeholders oppose these decisions (see Weiland 2016). The evidence cluster seems to rely on technical rationality as the key logic for arranging management. The salmon protection cluster promotes the view that those stakeholders with a normative position prioritise ecology, and salmon is capitalised before societal interests in top-down management (Weible et al. 2004). This cluster seems to rely on normative calls for ecological integrity as a management rationale. The traditional Sámi fishing cluster promotes recognition of local Sámi people as rightsholders who should have more rights than other actors (see also Larsen et al. 2017). Not only do the Sámi people on the Teno river have an economic stake in the salmon: their way of life is tightly intertwined with the salmon, they have a historical and cultural continuum with the Teno river and salmon, and their identity is also linked to salmon (Holmberg 2018; Kojo 1983; Turunen et al. 2020). These diverging perceptions of the kind of stakes that are at play, and who should have rights to decide on salmon, play a role in explaining the conflicts in belief between the four clusters, as well as conflicts in real life.

The difference between rightsholders and stakeholders concerns the relative position within the complex set of rights, histories, continuities, dependencies, obligations, and freedoms (Hiedanpää and Bromley 2013). These relations are complex, because different rights have different institutional support. For example, Sámi land and water rights in Finland do not have the same legal standing as they do in Norway, where the ILO 169 agreement has been ratified. Furthermore, the right to be secure from the negative impacts of other economic activities (e.g., salmon fishing) and administrative decisions add elements of culture and identity to the complex relations of rights. Indigenous and local communities are recognised as rightsholders by the UN Permanent Forum on Indigenous Issues (UN PFII 2016). Additionally, the International Union for Conservation of Nature’s (IUCN) policy on conservation and human rights recognises that indigenous and local communities are not mere stakeholders, but rightsholders to whom implementing agencies have statutory obligations (IUCN 2012, also Larsen et al. 2017; Wiessner 2011). The legal right to fish salmon is tied to real estate ownership along the river, but in 2019, the district court decided that several Sámi people in Utsjoki were entitled to fish on the Sámi homeland without the permission from Metsähallitus (the Forest and Park Service) that is usually required (Yle 2019). The case is now in the Supreme Court, and the decision concerning it will probably establish a precedent.

In our results, the traditional Sámi fishing cluster feel they should have more fishing rights than others. This is however questioned by the equal economic opportunity, salmon protection, and evidence clusters. The concepts of stakeholder and rightsholder can also capture the divergence between local Sámi fishers and external actors whose entire lifestyle and identity are not dependent on the right to fish. For rightsholders, fishing is not a mere hobby, but a constituent of identity and livelihood. Not only are current rights at stake: the foreseeable development of restrictions are—for whom, and by which measure and criteria.

### Identity and Struggle

Throughout the world, indigenous people struggle under the force of globalisation to ensure the continuance of indigenous culture and identity. These forces may take the form of cultural appropriation (Kuokkanen 2000) and ecological imperialism (Coates 2004). While indigenous identities are heterogeneous, their viability traditionally relies on access to lands and waters in their home territories (Oskal et al. 2009). Such access may be compromised by conservation legislation and legal practice to protect valuable environmental features (e.g., Dalhberg et al. 2010). Consequently, the cultural features and identities of indigenous and local communities may be compromised by government policy decisions (Persson et al. 2017).

Sámi hopes regarding land and water rights, self-determination, and concern for the continuity of traditional livelihoods like fishing and reindeer herding have introduced these contested issues to the public realm. In its moratorium against the Teno fishing agreement, the political and artistic activist group Ellos Deatnu has shown that the struggle has features of identity politics: the collective necessity to maintain the shield and simultaneously strengthen cultural resilience against outer pressures—in this case, in salmon administration and governance (Ellos Deatnu 2020; also Selfors 2015).

Human cultures and livelihood practices are often absent from natural scientific practice. Scientists operate according to their theoretical perspectives and research methods, and in the case of Teno salmon, they monitor the salmon population according to scientific standards and translate the information to fit administrative knowledge requirements. Teno salmon monitoring is funded by the Ministry of Agriculture and Forestry, and the Natural Resources Institute Finland (Luke), which is responsible for salmon monitoring, operates under the same ministry. Researchers are therefore strongly perceived as being more on the government side than that of locals. Indeed, the salmon administration and government scientists belong to the same institutionalised epistemic circle, which is not as broad as an epistemic community (Haas 2012) or as likeminded as an advocacy coalition (Sabatier 1998). However, they work in tandem, and are a good intellectual and practical fit, as our evidence-based decision-making cluster indicates.

From the Sámi perspective, this makes the government and scientists seem like colonial allies. Our results revealed that rather than being perceived as in objective pursuit of an equally accepted common good, science and administration players were not considered neutral actors. This often especially surprises scientists, but it also surprises administrators, who think they are pursuing the common good with the best law-based intentions and available evidence. Yet such tensions between science, administration, and indigenous people are not exceptional (Tuhiwai Smith 2012, p. 45–60). In contrast with the salmon protection and evidence-based decision-making clusters, the beliefs in traditional Sámi fishing cluster strongly advocates the view that indigenous knowledge can make significant contributions to sustainable environmental management (see also Tom et al. 2019).

Cochran et al. (2008, p. 22) take a normative stance, noting that researchers *“must continue to resolve conflict between the values of the academic setting and those of the community”* by considering how knowledge is embedded in indigenous communities and worldviews. This reflects the traditional Sámi fishing cluster views, which appear to propose that the science supporting administrative planning and decision-making must change. Despite the historical burdens between salmon scientists and local Sámi fishers, recent development and interaction has been positive, and scientists are today seen as more neutral knowledge providers than they were decades ago. This is due to the development of participatory practices and the long history of interactive scale sample collection (Turunen et al. 2020).

These contradictions also explain why the Sámi, as heterogenous indigenous communities, demonstrate an active willingness to participate in the politics of identity (Honig 2009; Connolly 1995). The politics of identity arises from a social struggle and feelings of social injustice. To cope with the power asymmetry between science and administration on the one hand and indigenous and local people on the other, Sámi concerns could be promoted by *“Indigenous articulations… where Indigenous peoples self-determine representations of their identities and interests”* (Diver 2017, p. 1). Allowing such articulations could level up the recognition of Sámi culture and rights in comparison to salmon protection and ecological sustainability. Indeed, the Finnish government has already reacted to the documented injustice, launching a truth and reconciliation process on Sámi issues (Juuso 2018). In a recent article that seeks the legitimacy of this process, the Teno fishing agreement of 2017 is seen as part of a settler-colonial policy in Finland among other cases (Kuokkanen 2020).

### Confidence and Respect

According to the Australian Health Ministers’ Advisory Council (2004, p. 7), cultural respect is “recognition, protection and continued advancement of the inherent rights, cultures and traditions” of indigenous people. Respect may be perceived differently by indigenous and non-indigenous people. In the Australian context, indigenous respect is about the equality of all, incorporating ancient law, philosophy, and spirituality, informing an appropriate code of conduct (AIATSIS 2019). Non-indigenous respect may be more individual and even commodified, and relies on the need to prove the worthiness of the one who is respected. Respect (and disrespect) may also be related to indigenous self-governance arrangements (UN 2018) and different indigenous and traditional knowledge systems (Tuhiwai Smith 2012, pp. 98–110). While the concept of respect is here viewed at a cultural level and manifested in the belief clusters in varying ways, the concept of confidence is linked to relationships between individuals and organisations, as well as institutional arrangements (Seligman 2007).

The Sámi and other locals in Utsjoki showed no confidence in salmon administration or salmon-related science, because the cultures, historic burdens, and epistemic practices differed so greatly (see also Heikkinen et al. 2010). In the traditional Sámi fishing cluster, science and administration represent colonialism by default, and even with long term interactions, no overall confidence has emerged. Possible reasons for this are that science and administration consider the Sámi as stakeholders instead of rightsholders, and that the identity struggle and quest for self-determination require the drawing of sharp distinctions between us and them.

Yet confidence and respect are linked to our findings on the colonialism of science and administrative practices. Strong beliefs about colonialism, as revealed by the Q exercise, imply that the Sámi people do not consider science and administration benevolent (that they seek to do good for the Sámi people) (on benevolence, see Mayer et al. 1995, pp. 718–719). The evidence-based decision-making cluster respects the Sámi, but the traditional Sámi fishing cluster indicates disrespect for salmon researchers. The reasons for this disrespect and even contempt are unclear. One reason may be that the process of giving scale samples is a one-way affair and thereby lacks reciprocity, which is a major way of building trust (see Ostrom and Walker 2005). We also believe there is a connection with Sámi culture and social circumstances. Relations of trust and respect are personal and communal, and depend on connections, not on formal positions, mandates, and protocols as in confidence (Pirson and Malhotra 2011). The Sámi have been colonised by different states, religions, and education systems (Lehtola 2015). Confidence in science and administration, and trust in scientists and administrators are not easy to build when they have been questioned for so long.

Not everything can be explained by the past and post-colonialism. Many scientists focus on environmental sustainability, seeing sustainability as a moral imperative that justifies the compromising of Sámi rights (see Wilshusen et al. 2002; Nygren 2013). Actors with such beliefs may therefore consider it risky to trust Sámi people in decision-making, because they believe it leads to decisions that compromise ecological sustainability. Because the stakes are high and the rights conflict, the level of perceived risk in trusting the other party is also high, making those who are trusting vulnerable (e.g., Stern and Coleman 2015; Möllering 2001). The case is further complicated, because the Teno salmon are affected by various other drivers in addition to Sámi fishing.

In trustworthy, confident, and respectful salmon policy planning and administrative decisions, facts ought to find a home, fit formal decision-making procedures and protocols, and constitute lawful administrative decisions. It is in the interest of the administration to ensure the lawful design and implementation of management rules, because decisions need to hold up in legal reviews. The Teno agreement has partly failed in this respect. Scientific knowledge of salmon has been considered, but the district court judgement indicates that traditional fishing rights have not carried weight in decision making (Virolainen 2019). However, it remains to be seen how the judgement holds in other judicial decrees.

## Concluding Remarks

Although our case is specific to the Teno river, by using SOM and Q methodology we were able to come to some general insights on stakes and rights, identity and struggle, and confidence and respect that are likely to be relevant for other cases of environmental governance in indigenous lands and waters. The discussed themes were not pre-known, but they were made visible—that is, pragmatically abducted—by the SOM-based statement clustering. The Q method is not immune to bias. Our precautionary measure was that all authors participated in the statement design and the interpretation of heatmaps. We therefore hope the potential for bias was decreased, and the validity of results and insights was increased.

All the webs of belief constitute different epistemic grounds and provide different understandings of the significance of the Teno salmon. It is necessary to acknowledge these differences to achieve feasible policy goals and management measures. However, as local and indigenous beliefs diverge significantly from scientific knowledge and administrative knowledge requirements, the normative aim of policy should be to develop institutional and cultural sensitivity to the multitude of different positions and the consequent institutional arrangements, administrative knowledge requirements, and governance practices to ensure that traditional and local interests and knowledge—true and circumstantially functioning beliefs—also fit protocols and routines, without being problematic from the administrative law perspective.

The struggle to find sustainable solutions starts with knowledge, but as we have shown, it is also connected to an entire set of identities, stakes, and rights that greatly complicates natural resource decisions. We have increased the understanding, offering basic grounds for identifying problems and even finding solutions for the Teno and beyond. Our findings suggest that while salmon governance should continue to build on existing legislation and stakeholder interests regarding management objectives, it should also systematically focus on the habits of thinking constituting beliefs about the underlying legislation, interests, and management objectives. According to our results, beliefs understood as preparedness to act give concrete grounds for a perpetual quest for trust, confidence, and respect.

Salmon knowledge, whether it is traditional, local, or scientific, concerns facts about salmon—the catch, lifecycle features, and their changes. Yet Sámi beliefs about salmon do not really build on the same ground as the administration. Salmon has an immense cultural value for the Sámi; it is “ethnic property”. Science also has its blind spots or less studied topics, such as the significance of the broader environment and salmon predators, and the nature and significance of indigenous Sámi property rights. However, this is the fault of the entire institutional set-up, which defines policy problems and sets the decision criteria, not that of science or individual scientists.

Salmon management in the Teno has been a top-down regulatory activity. Recent regional developments indicate that the conditions for adaptive policy and management have become more favourable. The regional administration has established a local monitoring group to report on changes in Teno salmon management. In addition, the Fishing Act (379/2015) orders every fishery region to prepare a management plan. The Teno fishery region started the process in 2020. As the salmon planning and decision making become more participatory, it may be that the interplay between local communities, science, and government will develop more quickly. It is to this interplay that our research seeks to contribute, both theoretically and practically.

## Appendix 2. Q Statements

1. The Teno salmon study will take into consideration the sustainability of ecological, social, economic and cultural aspects in a balanced way.
2. Salmon scientists and people deciding salmon issues consider the knowledge and data in the same way.
3. The Teno salmon research focuses too much on the salmon as an animal, rather than the wider river ecosystem.
4. Salmon research focuses too much on fishing pressure on the stocks of salmon and underestimates the impacts of predators.
5. The scientific salmon knowledge is impartial.
6. Salmon scientists are independent.
7. Salmon scientists respect the Sámi culture.
8. Salmon scientists make use of traditional salmon knowledge.
9. The results of salmon research will be used against locals in the decision-making.
10. Salmon scientists represent colonialism.
11. Research knowledge affects little to salmon policy.
12. Traditional knowledge must be taken into account in the salmon policy to maintain Sámi culture.
13. Scientific knowledge alone is not enough to support salmon policy, traditional knowledge is also needed.
14. Research has gained local understanding of salmon stocks.
15. A new fishing agreement was necessary to revive salmon stocks in tributary streams of rivers Teno.
16. Traditional Sámi salmon knowledge is no longer valid because of altered natural conditions.
17. Salmon decisions should be based on scientific knowledge rather than traditional knowledge.
18. It is difficult to make use of traditional knowledge in decision making.
19. Decision-makers take recent scientific knowledge into account enough when making decisions.
20. Sámi traditional salmon knowledge is different from the rest of the local salmon knowledge.
21. Decision-makers take the needs of Sámi people into account enough when making decisions on the salmon.
22. The lobbying will greatly affect salmon policy.
23. In salmon management planning it is difficult to have the balanced consultation of stakeholders, since confirmed policy determines how to proceed.
24. Decision making concerning Teno salmon issues should be transferred to the Sámi people by developing the self-regulation of Sami home district.
25. The current salmon management respects the Sámi culture.
26. The current salmon management represents colonialism.
27. Non-Sámi needs should be taken into account more in salmon stock management.
28. Norway’s salmon policy at River Teno hinders planning and decision making on the Finnish side of the river.
29. Traditional fishing is a way of life and a livelihood.
30. Traditional fishing is a hobby.
31. The Sámi people are fishing in a sustainable way.
32. The Sámi people promote their issue in inappropriate manners.
33. The Sámi people are losing their traditional salmon knowledge because of poor salmon management.
34. Sámi as indigenous people should have more extensive fishing rights than others.
35. Tourist fishing should be limited the same in relation to the Sámi people’s traditional fishing.
36. Only the Sámi should sell fishing licenses on Teno River.
37. Fishers’ interests in the Teno River are essentially parallel.
38. Different stocks in tributary streams have no significance for fishing.
39. Non-local landowners should have the right to fish with nets.
40. The continuity of tradition should be taken into account in the decision-making process.
41. Utsjoki-born people (now living outside of Utsjoki) should have the same fishing rights as local people.
42. All fishing groups should have unambiguous catch quotas.
43. The current Teno agreement should be rejected and new negotiations should start, since the rights of the Sámi had too little attention.

## Footnotes

1 Finland and Norway have jointly regulated fishing in the Teno since 1873. Before the Teno Fishing Agreement of 2017, the regulation was modified nine times (1920, 1938, 1953, 1961, 1973, 1979, 1983, and 1990) (Anti 2015, p. 11; Länsman 2014).

2 Northern Sámi was the language in 32 interviews, and Finnish in 5. Two interviews were pair interviews. Informants were selected from a group of participants who had been involved in a 45-year collaboration between the Natural Resources Institute Finland (Luke) (until 2015 Finnish Game and Fisheries Research Institute, RKTL) and local fishers in the region (Niemelä et al. 2005). These informants had provided salmon scale samples to Luke. Second, further informants were chosen based on snowball sampling (e.g., Silverman 2013) by first asking the informants and other locals about people with a good knowledge of traditional salmon fisheries.
